# Enhanced Performance of PbS-quantum-dot-sensitized Solar Cells via Optimizing Precursor Solution and Electrolytes

**DOI:** 10.1038/srep23094

**Published:** 2016-03-15

**Authors:** Jianjun Tian, Ting Shen, Xiaoguang Liu, Chengbin Fei, Lili Lv, Guozhong Cao

**Affiliations:** 1 Institute of Advanced Materials and Technology, University of Science and Technology Beijing, Beijing, 100083, China; 2 Beijing Institute of Nanoenergy and Nanosystems, Chinese Academy of Sciences, 100083, China; 3 Department of Materials and Engineering, University of Washington, Seattle, WA, 98195-2120, USA

## Abstract

This work reports a PbS-quantum-dot-sensitized solar cell (QDSC) with power conversion efficiency (PCE) of 4%. PbS quantum dots (QDs) were grown on mesoporous TiO_2_ film using a successive ion layer absorption and reaction (SILAR) method. The growth of QDs was found to be profoundly affected by the concentration of the precursor solution. At low concentrations, the rate-limiting factor of the crystal growth was the adsorption of the precursor ions, and the surface growth of the crystal became the limiting factor in the high concentration solution. The optimal concentration of precursor solution with respect to the quantity and size of synthesized QDs was 0.06 M. To further increase the performance of QDSCs, the 30% deionized water of polysulfide electrolyte was replaced with methanol to improve the wettability and permeability of electrolytes in the TiO_2_ film, which accelerated the redox couple diffusion in the electrolyte solution and improved charge transfer at the interfaces between photoanodes and electrolytes. The stability of PbS QDs in the electrolyte was also improved by methanol to reduce the charge recombination and prolong the electron lifetime. As a result, the PCE of QDSC was increased to 4.01%.

Like other traditional photovoltaics, p-n junction crystalline silicon (c-Si) solar cells are costly to manufacture and install. Developing highly efficient solar cells that would be economically viable is becoming an increasingly urgent task imposed on the scientists around the world^1^,^2^. Semiconductor quantum dots (QDs) are solution-processable semiconductor nanocrystals, and they have been recognized as a suitable material for next-generation solar cells due to their versatile optical and electrical properties^3^,^4^,^5^. Recently, QDs have been used in photovoltaics to form quantum dots solar cells (QDSCs) for the following reasons: (1) tunable band gap depending on QD size, (2) large extinction coefficient, (3) stability toward water and oxygen, and (4) multiple exciton generation (MEG) with single-photon absorption^6^,^7^,^8^. The theoretical power conversion efficiency (PCE) of QDSCs can reach up to 42% in view of MEG effect of QDs, which is much higher than that of semiconductor solar cells (31%) according to Schockley-Queisser limit^9^,^10^,^11^.

Among the various QDs, CdS, and CdSe are usually used for QDSCs because of their high potential for light harvesting in the visible light region^12^,^13^. In addition, CdS can be used to induce CdS/CdSe co-sensitization, which broadens the optical absorption of QDs and increases the efficiency of the solar cells. Recently, a CdS/CdSe QDSC with efficiency of 6.33% was prepared by doping Mn^2+^ into CdSe^14^. Kamat *et al*. reported a PCE of 6.6% CdS/CdSe QDSC after the introduction of a layer of Cu_x_S on the surface of CdSe QDs^15^. However, CdSe has a band gap (E_g_) of 1.7 eV and may absorb photons with wavelengths shorter than 700 nm^16^. Using these QDs (E_g_ > 1.5 eV), it may not be possible to utilize the full solar spectrum, which limits improvement of the photocurrent density and PCE of the solar cells^17^. Low-E_g_ (<1.5 eV) QDs have shown considerable potential for the QDSCs with high photo-generated current density and PCE attributable to near infrared (IR) absorption. PbS is one of the most intensively studied low E_g_ semiconductors, with an E_g_ in the near IR region of 0.41 eV^17^,^18^. PbS also has a high absorption coefficient of 1–5 × 10^5^ cm^−1^ and Bohr excitation radius of 18 nm^19^,^20^. In this way, PbS QDs is an ideal sensitizer for QDSCs. However, PbS-sensitized solar cells are much less efficient than CdS/CdSe QDSCs, no more than 4% without co-sensitization or doping. A great deal of effort has been made to enhance the PCE of QDSCs^18^,^21^,^22^,^23^. Mora-Seró *et al*. developed a CdS coating layer that could be added to the surface of PbS to produce PbS-CdS co-sensitized solar cells^18^. The PCE of the solar cells exceeded 4%. N.G. Park *et al*. reported a highest PCE of 5.6% for another set of solar cells, which was produced by doping Hg into PbS^17^. Without using co-sensitization or doping, the highest PCE of any solar cell was 3.6%, and it was reported by Loiudice and Rizzo^24^. To date, the PCE of PbS QDSCs do not reach expectations. This may be because of the high charge recombination of the solar cells and instability of PbS in the electrolyte^18^,^21^,^25^. To solve these problems, research should focus on controlling the synthesis process for QDs of suitable quantity and size, and optimizing the electrolyte solution to improve their stability.

In a typical fabrication of QDSC photoanodes, QDs are deposited directly onto the oxide anode in the precursor solutions^9^,^26^. These approaches, including chemical bath deposition (CBD)^27^ and successive ionic layer absorption and reaction (SILAR)^28^,^29^, have shown good performance because of the close contact between QDs and oxide anodes. CBD is a relatively simple method of depositing QDs and nanoparticles. It has many advantages, such as stable yield, robust adherence and good reproducibility. The growth of QDs is heavily dependent on the growth conditions, such as the duration of deposition, the composition and temperature of the solution, and the characteristics of the anode films^30^. At present, CBD is rarely used in the fabrication of PbS QDs for the solar cells. The SILAR method is based on successive reactions on the surface of oxides. Each reaction is followed by rinsing, which facilitates a heterogeneous reaction between the solid phase and the solvated ions in the solution^30^. The mechanism underlying SILAR shows that the number and sizes of adsorbed QDs affect the performance of QDSCs. These factors have a close relationship with the concentration and composition of the precursor solution. Although the SILAR is widely used in PbS-based QDSCs, there are few studies on the mechanism underlying the relationship between PbS growth and the concentration of the precursor solution. Here, the SILAR approach was used for the fabrication of PbS QDs, and the growth, quantity, and size of PbS QDs were studied. In addition, to improve the diffusion and wettability of the electrolyte in the mesoporous oxide film, 30% of the deionized water of polysulfide electrolyte was replaced with methanol. The stability of PbS in the electrolyte was also improved by adding methanol. Results showed that the solar cells could be optimized by adjusting precursor concentration and electrolyte, producing PCEs of up to 4.01%.

## Results

**Figure 1:**
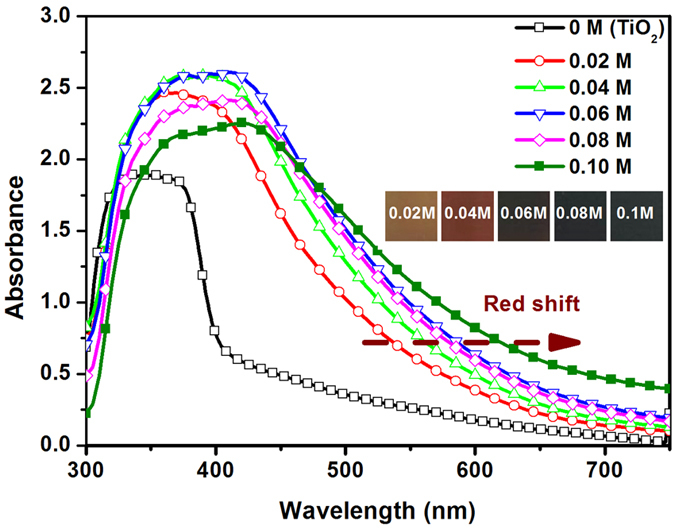
UV-visible spectra curves of the bare TiO_2_ film and TiO_2_ films loaded with PbS QDs from precursor solutions of different concentrations; inset shows the color of the film.

PbS QDs were directly deposited on mesoporous TiO_2_ films using successive ionic layer adsorption and reaction (SILAR). The concentration of the precursor solution was found to be a key factor affecting the quantities and particle size of QDs. Although there are no ideal measurements that can be used to estimate the numbers and particle sizes of PbS within the film accurately because of the low mass fraction and very small size of QDs, the effects of the concentration can be deduced by measuring the changes in the light absorbance of film loaded with PbS QDs. Figure 1 displays the UV-visible spectra curves of the bare TiO_2_ film and the films loaded with PbS QDs deposited from precursor solutions with different concentrations. It is clear that both absorbance and absorption range of the TiO_2_ film are increased by loading PbS QDs. In addition, the absorbance of the films increases as the increase of concentration, and peaks at a concentration of 0.06 M. However, when the concentration is increased further, the absorbance of the film decreases. The number of QDs is proportional to the absorbance of the film with loading of QDs. For this reason, the highest number of QDs is synthesized in 0.06 M precursor solution. As shown in the inset of Fig. 1, the films gradually darken as the concentration increased. When the concentration of the solution reaches 0.06 M, the film is entirely black. As shown in Fig. 1, results also show that the absorption edges of the film loaded with PbS QDs shift towards long wavelengths as the concentration increased (red shift), which causes a decrease in the band gap (E_g_). The decrease in E_g_ is caused by increases in the size of QDs (quantum size effect). The PbS QDs increased in size as concentration increased. To understand the relationship between the concentration and optical absorption properties of QDs, the formation and growth of QDs should be discussed further.

**Figure 2:**
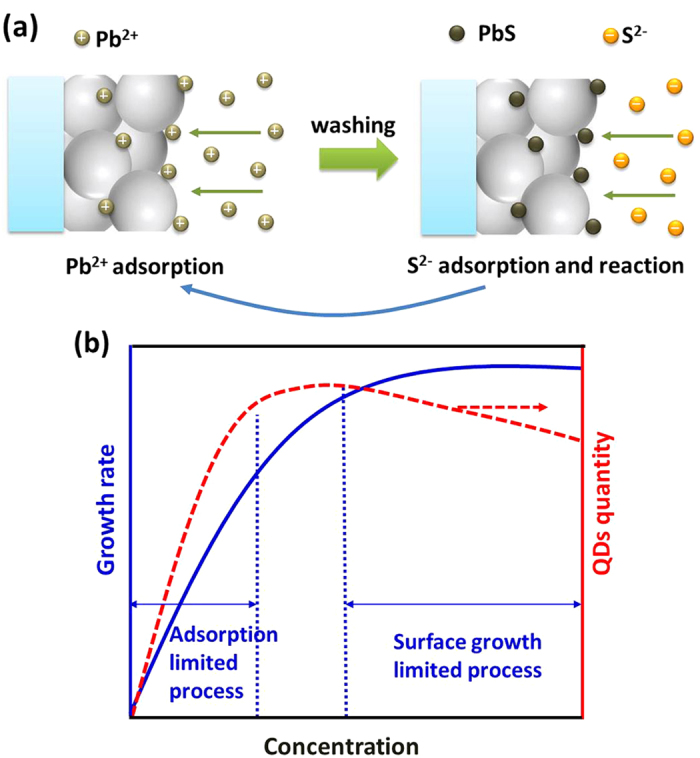
(**a**) Schematic synthesis of PbS QDs using the SILAR method and (**b**) the effect of precursor concentration on the growth rate and quantity of PbS QDs. It is derived from the findings of a previous study^31^.

Figure 2(a) shows the synthesis of PbS QDs using the SILAR method. Pb^2+^ ions are adsorbed onto the surface of oxide (TiO_2_) nanoparticles during the first dipping step in the metallic precursor solution. After the washing, Pb^2+^ reacts with S^2−^ to form PbS QDs on the TiO_2_ surface at the second dipping step in the sulfur precursor solution. When a new phase forms on the surface of another material, the process is called heterogeneous nucleation^31^. Figure 2(a) shows that the formation of PbS QDs involves heterogeneous nucleation. After nucleation, PbS crystals grow quickly. Crystal growth can be generally considered a heterogeneous reaction. The concentration of the precursor solution is key factor for the growth rate. Figure 2(b) shows the effect of the concentration on the growth rate of PbS QDs. This effect is derived from the findings of a previous study according to the fundamentals of heterogeneous nucleation and crystal growth^31^. When the concentration of the precursor solution is low, the adsorption of ions acts as a rate-limiting factor for crystal growth. For a given system, the growth rate of the crystal increases with the increase of the concentration in a linear fashion. Further increases in the concentration can cause a change in the limiting factor from the adsorption of ions to the surface growth of the crystal. When the surface growth becomes a limiting factor, the growth rate of the crystals becomes independent of the concentration. As shown in Fig. 2(b), when the adsorption of ions is the limiting factor, both the number of QDs and particle size increase as the concentration of the precursor solution increases because more ions can be adsorbed accordingly. When the concentration exceeds 0.06 M, the number of ions adsorbed reaches saturation and the surface growth become the limiting factor. The number of ions adsorbed does not increase with further increases in concentration. However, the crystals still increase in size. These large crystals can block some interior pores, which reduces the number of adsorbed ions. The number of PbS QDs decreases slightly, which is closely consistent with the results of UV-visible absorbance.

**Figure 3:**
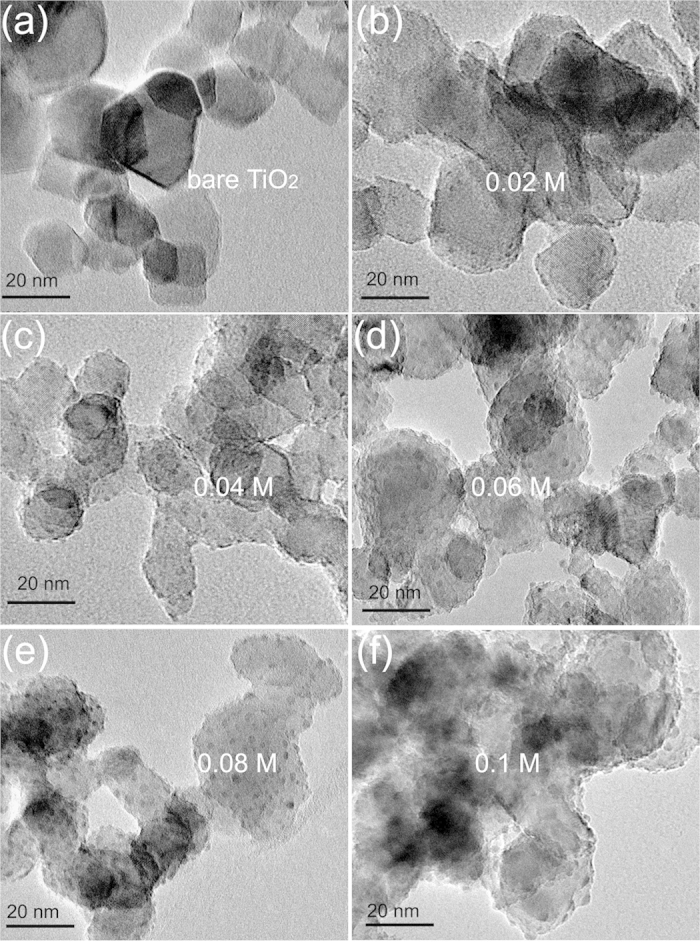
TEM images of (**a**) bare TiO_2_ film, and TiO_2_ film loaded with PbS QDs from precursor solutions of different concentrations (**b**) 0.02 M, (**c**) 0.04 M, (**d**) 0.06 M, (**e**) 0.08 M and (**f**) 0.1 M.

Figure 3 shows the TEM micrographs of bare TiO_2_ nanoparticles and PbS QDs adsorbed TiO_2_ nanoparticles prepared using precursor solutions of different concentrations. The TEM samples were selected from the small TiO_2_ nanoparticles adsorbed with PbS QDs, which were suspended in the ethanol. Although TEM is not an ideal approach to determining the size and number of PbS QDs at the different precursor solution concentrations, it can serve as a suitable supplementary method. The results recorded here clearly show that semi-spherical QDs form only sparsely on the surfaces of TiO_2_ nanoparticles. Both the number and size of QDs increase with increasing precursor solution concentration. The average size of QDs is calculated by statistical analysis of TEM images. The average sizes of QDs are 2.44 ± 0.23 nm for Fig. 3(b), 3.93 ± 0.27 nm for Fig. 3(c), 4.72 ± 0.31 nm for Fig. 3(d), 4.93 ± 0.34 nm for Fig. 3(e) and 5.36 ± 0.38 for Fig. 3(f), respectively. The concentration exceeds 0.06 M, and the size and number of QDs show no obvious change, which is consistent with findings shown in Fig. 2(b). However, the concentration of precursor solution is increased further (up to 0.1 M), QDs are easy to aggregate, which blocks the holes and pores among the TiO_2_ nanoparticles as shown in Fig. 3(f). The aggregation of QDs will reduce the PCE of the solar cell due to high surface charge recombination. In addition, the size of the PbS QDs is an important parameter that may regulate charge injection. According to results reported by Braga *et al*.^32^ and Concina *et al*.^33^, the conduction band (CB) of bulk PbS is lower than that of TiO_2_, which suppresses electron injection from PbS into TiO_2_. This problem can be solved by reducing the size of PbS particles to below the Bohr’s radius due to the quantum confinement. The energy position of CB is up-shifted by decreasing the size of PbS. So the fast electrons can transfer from PbS into TiO_2_. However, very small QDs have many more surface defects, which cause high surface charge recombination. For this reason, PbS QDs should be neither too large nor too small.

**Figure 4:**
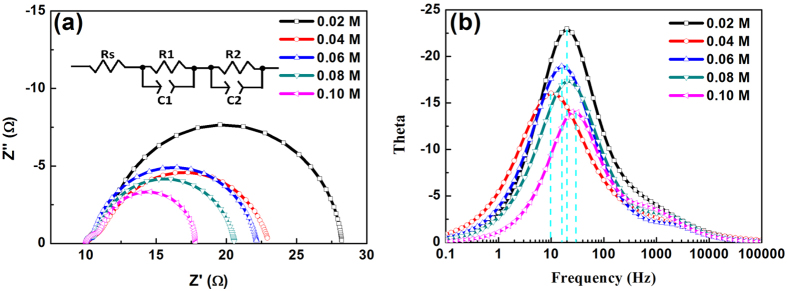
(**a**) Nyquist plot and (**b**) Bode plot curves of the QDSCs under light intensity, 100 mW/cm^2^.

Figure 4 shows the electrochemical impedance spectra (EIS) of QDSCs under the light intensity of 100 mW/cm^2^. The fitting results of the impedance spectra are listed in Table 1. Under illumination, the solar cell can be considered a diode^34^,^35^,^36^,^37^. As shown in Fig. 4(a), the hemisphere in the high-frequency region (10^4^–10^2^ Hz) represents the resistance of charge transport at the counter electrode/electrolyte interface (R_1_). At low frequencies, the impedance related to the charge transport at the TiO_2_/PbS/electrolyte interface can be described using R_2_. The lower R_2_ is, the faster the charge transfer and transport at the TiO_2_/PbS/electrolyte interface is. The present work is focused mainly on the R_2_, which shows the effect of precursor solution concentration on the charge transfer and transport at the TiO_2_/PbS/electrolyte interfaces. Results show that R_2_ decreases gradually with increasing precursor concentration. This is probably due to the increase in the number and size of QDs. In this way, the charge transport is sped up by increasing the concentration of the precursor solution, which facilitates charge collection for a high photo-current density of the solar cell. However, R_2_ is also considered as a resistance of charge recombination at the interfaces of TiO_2_/QDs/electrolytes^38^. Decreases in R_2_ can boost the charge recombination and shorten electron lifetime, which causes the performance of the solar cell to deteriorate. Figure 4(b) shows the Bode plots of the QDSCs. The curve peak of the spectrum can be used to determine the electron lifetime according to the equation (1)^39^.


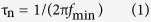


Here, τ_n_ is the electron lifetime, *f*_min_ is minimum frequency of the Bode plot curves. The corresponding results are listed in Table 1. As shown, τ_n_ increases as the concentration of the precursor solution increases. The τ_n_ is up to the maximum value (12.6 mS), when the concentration is 0.04 M. When the concentration exceeds 0.06 M, τ_n_ decreases visibly. This may be because of increases in charge recombination in high-concentration solutions (>0.06 M). According to the results of the electron transfer and lifetime, the appropriate precursor concentration is around 0.06 M, which is in consistent with the results of the absorbance (Fig. 1).

**Figure 5:**
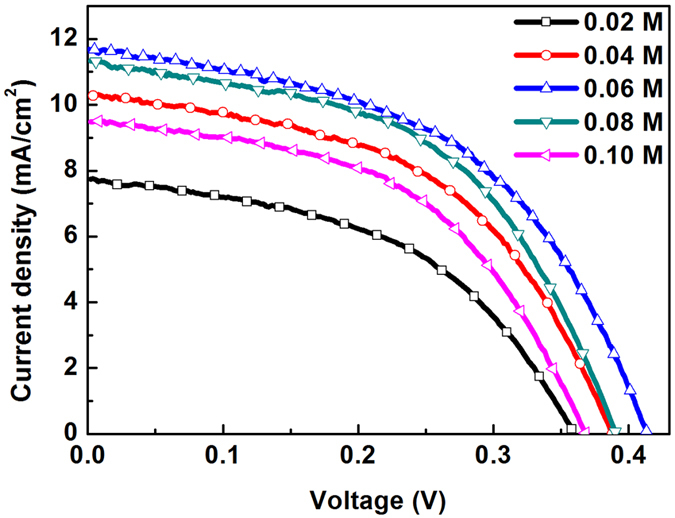
Photocurrent density-voltage (J-V) curves of QDSCs measured under AM 1.5 G, at 1 sunlight intensity.

Figure 5 shows photocurrent density-voltage (J-V) characteristics of solar cells measured under the illumination of one sun (AM 1.5, 100 mW·cm^−2^). The performance parameters of the solar cells, including open circuit potential (V_oc_), short circuit current density (J_sc_), fill factor (FF), and power conversion efficiency (PCE), are listed in Table 2. As shown, all parameters of J_sc_, V_oc_, and PCE increase as the concentration of the precursor solution increases from 0.02 M to 0.06 M. The increase in J_sc_ is caused by increases in the number of PbS QDs, which cases more optical absorption and faster electron transfer. However, when the concentration increases further to 0.08 M, J_sc_ decreases slightly due to the decrease in light absorbance and electron collection. In view of the EIS results, the decrease in V_oc_ can be attributed mainly to the increased surface charge recombination and shortened electron lifetime. As a result, the highest PCE, 2.41%, is obtained when the concentration of the precursor solution is 0.06 M.

**Figure 6:**
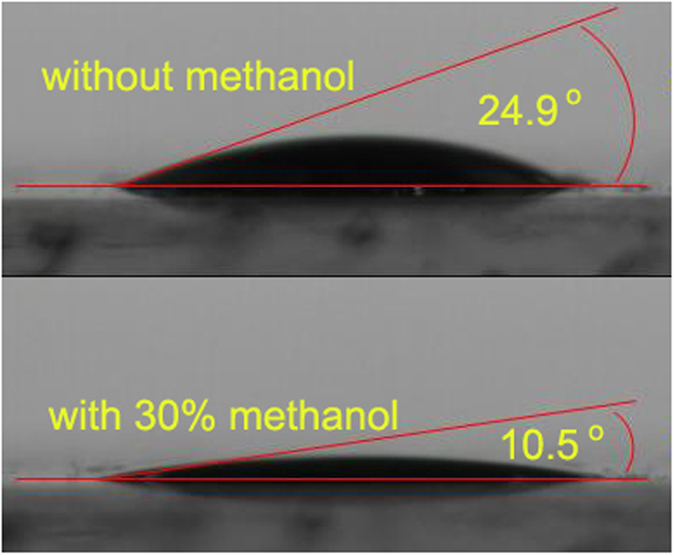
Contact angle images showing the wettability of the electrolyte with and without methanol.

**Figure 7:**
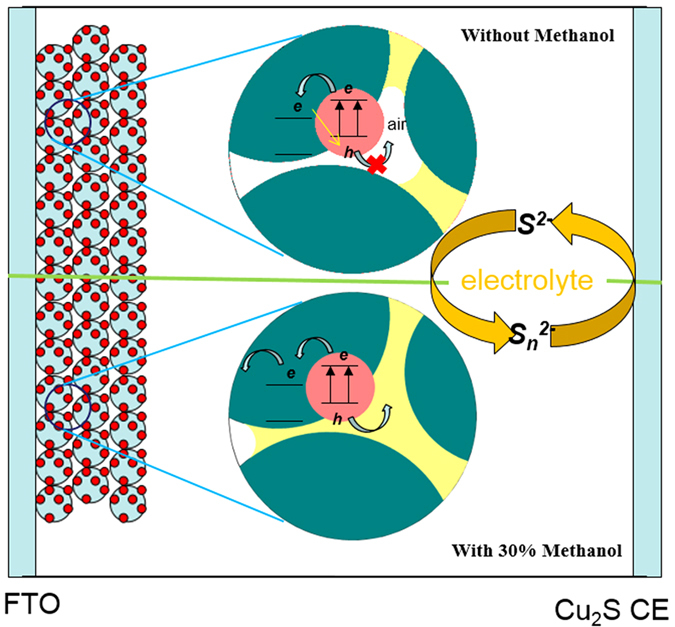
Schematic of the wettability and diffusion of different electrolytes.

To improve the wettability and penetrability of the electrolyte, methanol was replaced with 30% deionized water of polysulfide. As shown in Fig. 6, the contact angle of the electrolyte with methanol is 10.5°, which is much smaller than that of the electrolyte without methanol (24.9°). The smaller the contact angle is, the better the wettability of the electrolyte is. Figure 7 shows an illustration of the wettability and diffusion of the different electrolytes. Because there are a large number of adsorbed air molecules on the surface of TiO_2_ nanoparticles, the interior pores of the film cannot be filled with the electrolyte solution. The improvement of wettability increases the penetrability of the electrolyte in the film, which is also helpful to the redox couple diffusion in the electrolyte and charge transport at the interfaces between photoanodes and electrolytes. In addition, PbS QDs is not stable in contact with polysulfide electrolyte, which decreases the photo-current density of the solar cell^25^. The methanol in the electrolyte is found to improve the stability of PbS QDs in the electrolyte, and thus increase the photo-current density of the solar cell. To evaluate the properties of the electrolyte and stability of PbS in the electrolyte, the EIS of the solar cells should be studied further.

**Figure 8:**
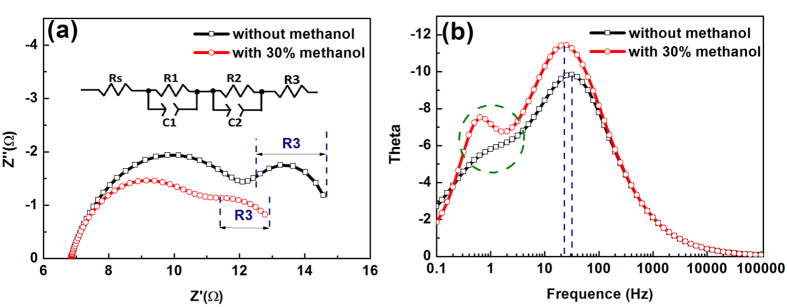
(**a**) Nyquist plot and (**b**) Bode plot curves of the QDSCs under light intensity, 100 mW/cm^2^.

Figure 8 shows EIS curves of the QDSCs under a light intensity of 100 mW/cm^2^. As shown in Fig. 7(a), the R_2_ of the solar cells using the electrolyte with 30% methanol is much lower than that of the one without methanol. It means that the electron transfer at the TiO_2_/PbS/electrolyte interface is accelerated by adding methanol to the electrolyte. The hemisphere in the low-frequency region (0.1–10 Hz) represents the diffusion impedance within the electrolyte^40^. The improved electrolyte smaller value of R_3_ than electrolyte without added methanol, indicating that the rate of diffusion of the redox couple in the electrolyte faster. As shown in Fig. 8(b), in the region of 0.1–1 Hz, there is an obvious peak in the red line Bode curve. This means that the improved electrolyte responds better to the change in frequency than the previous electrolyte. In this way, electron transfer and transport both at the interfaces and within the electrolyte solution can be improved by adding methanol to the electrolyte. In addition, the electron lifetime of the improved electrolyte is longer than that of the unmodified electrolyte in view of the peak of the Bode curve shift to low frequency. The increase in electron lifetime is derived from the decrease in the surface charge recombination, which can be attributed mainly to improvement in the stability of PbS in the electrolyte. In addition, the methanol in the electrolyte solution may be involved in the redox reaction in the solar cell, which is another possible way to improve the performance of the device. However, it is very difficult to find evidence to support this conclusion.

**Figure 9:**
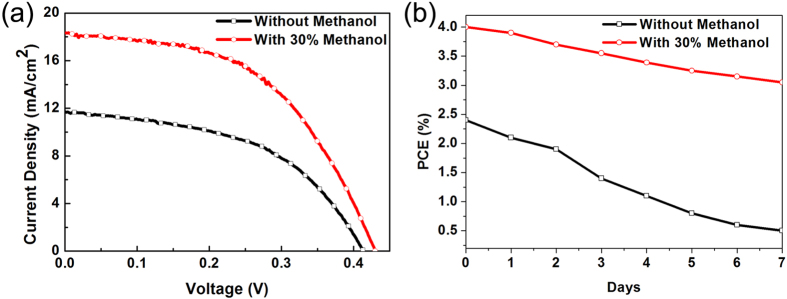
(**a**) Photocurrent density-voltage (J-V) curves of QDSCs measured under AM 1.5 G, at 1 sunlight intensity and (**b**) efficiency of QDSCs after different days when the sealed cells were exposed to air at 24 °C.

Figure 9(a) shows photocurrent density-voltage (J-V) characteristics for the solar cells measured under the illumination of one sun (AM 1.5, 100 mW ∙ c^−2^). The performance parameters of the solar cells are listed in Table 3. The solar cell with the improved electrolyte exhibits a high performance: J_sc_ = 18.34 mA/cm^2^, V_oc_ = 0.43, FF = 50.86%, PCE = 4.01%. As shown, J_sc_ of the solar cell is visibly improved by adding methanol to the electrolyte, which is the main cause of improvement of the conversion efficiency. The remarkable increase in J_sc_ can be attributed to the improvement of PbS stability and to the enhancement of the transfer and lifetime of the electrons. Like J_sc_, V_oc_ is increased by adding methanol to the electrolyte. This may have been due to the decrease in the charge recombination. As a result, the PCE of the solar cell is 66% higher than that of the solar cell made without added methanol (2.41%). The PCE recorded here, 4.01%, is one of the highest values for PbS-quantum-dot-sensitized solar cells without using co-sensitization or doping. In addition, the methanol in the electrolyte improved the stability of the solar cells as shown in Fig. 9(b).

## Discussions

PbS quantum dots (QDs) were directly deposited onto the mesoporous TiO_2_ film using the SILAR method. The process of formation of PbS QDs on the surfaces of TiO_2_ nanoparticles is heterogeneous nucleation, which is visibly affected by the concentration of the precursor solution. The appropriate number and size of QDs within the TiO_2_ film were determined when the concentration was 0.06 M. As the concentration of precursor solution increased further (>0.06 M), the properties of QDSC started dropping due to the increase in surface charge recombination. To improve the wettability and penetrability of the electrolyte, the 30% deionized water of polysulfide electrolyte was replaced with methanol. The electrolyte not only accelerated the redox couple diffusion in the electrolyte and the charge transfer at the interfaces between photoanodes and electrolyte but also enhanced the stability of PbS in the electrolyte to reduce the surface charge recombination. This produced a PCE of 4.01%, which is one of the highest values ever recorded for PbS QDSCs without co-sensitization or doping.

## Methods

### Preparation of mesoporous TiO_2_ films

Mesoporous TiO_2_ films with the thickness of 10 ± 0.5 μm were prepared via the doctor blading on the F:SnO_2_ glass (FTO, 8 Ω/square) substrates using TiO_2_ pastes mixed of TiO_2_ nanoparticles (Degussa P25), ethyl cellulose and α-terpineol^9^,^14^. The TiO_2_ films underwent a sintering process in air at 500 °C for 30 min.

### Fabrication of PbS QDs Sensitized Photoelectrodes

PbS QDs were deposited on the surface of TiO_2_ films using successive ionic layer adsorption and reaction (SILAR) method. The mesoporous TiO_2_ film was first dipped in a Pb(NO_3_)_2_ solution with solvent of deionized water and methanol as volume ratio of 1:1 for 1 min. Successively, the film was dipped into a Na_2_S solution with solvent of deionized water and methanol as volume ratio of 1:1 for 1 min to allow S^2−^ to react with the preadsorbed Pb^2+^, leading to the formation of PbS. Between each dipping, the electrode was thoroughly washed with methanol. The sequence of dipping metallic precursor-washing-dipping sulphur precursor-washing constitutes a SILAR cycle. In total, three cycles were employed to obtain a suitable amount of PbS on the TiO_2_ film. The precursor (Pb(NO_3_)_2_, Na_2_S) solution concentrations of 0.02 M, 0.04 M, 0.06 M, 0.08 M and 0.1 M were adopted to seek an appropriate concentration for the solar cells. After that, a ZnS passivation layer was deposited by two SILAR cycles while being soaked in an aqueous solution containing 0.1 M Zn(NO_3_)_2_ and 0.1 M Na_2_S, which act as Zn^2+^ and S^2−^ sources, respectively.

### Counter electrode and Electrolyte

The Cu_2_S films were used as the counter electrode in this study^14^,^41^,^42^, which were prepared on the brass foils as the following: the brass foils were immersed into 37% HCl at 70 °C for 20 min, then were washed and dried. The etched brass foils were dipped into aqueous solution containing 1 M S and 1 M Na_2_S to form a Cu_2_S film. The electrolyte employed in this study was composed of 1 M S and 1 M Na_2_S in deionized water or mixture of deionized water and methanol with volume ratio of 7:3.

### Materials and QDSC characterization

The absorption spectra were measured using a Shimadzu UV-3600 spectrophotometer. The morphology of the TiO_2_ nanoparticles absorbed quantum dots was characterized by transmission electron (TEM, Tecnai G2 F20) microscopy. The electrochemical impedance spectroscopy (EIS) was carried out with use of an impedance analyzer (ZAHNER CIMPS) under light intensity, 100 mW/cm^2^. Contact angles of the electrolytes were measured with the static sessile drop method using a VCA Optima goniometer. The photovoltaic characteristics of the solar cells were evaluated using simulated AM 1.5 sunlight with an output power of 100 mW/cm^2^ provided by a 3A grade solar simulator (Crowntech, SOL02 series). The active area of the QDSCs was 0.1256 cm^2^ determined by a photo-mask. The current-voltage characteristics were performed using an electrochemical workstation (ZAHNER, ZENNIUM). We prepared five samples for a certain type of solar cell. So the standard deviation of the all properties is based on the statistical data of five samples measured.

## Additional Information

**How to cite this article**: Tian, J. *et al*. Enhanced Performance of PbS-quantum-dot-sensitized Solar Cells via Optimizing Precursor Solution and Electrolytes. *Sci. Rep*. **6**, 23094; doi: 10.1038/srep23094 (2016).

## Figures and Tables

**Table 1:** Electrochemical impedance results of QDSCsa.

Concentration (M)	R_1_(Ω)	R_2_(Ω)	τ_n_ (mS)
0.02	1.5 ± 0.4	16.7 ± 0.7	8.0 ± 0.5
0.04	1.1 ± 0.2	11.9 ± 0.5	12.6 ± 0.9
0.06	0.4 ± 0.1	11.8 ± 0.7	10.1 ± 0.9
0.08	0.7 ± 0.1	9.9 ± 0.4	8.0 ± 0.8
0.10	1.1 ± 0.2	6.8 ± 0.4	6.3 ± 0.6

^a^The standard deviation of the properties is based on the statistical data of five cells.

**Table 2:** Photovoltaic properties of QDSCs from J-V curves^a^.

Concentration (M)	*V*_oc_(V)	*J*_sc_(mA/cm^2^)	FF(%)	PCE(%)
0.02	0.36 ± 0.01	7.44 ± 0.23	48.40 ± 2.13	1.34 ± 0.10
0.04	0.39 ± 0.02	10.32 ± 0.31	49.46 ± 1.44	1.98 ± 0.13
0.06	0.41 ± 0.02	11.68 ± 0.36	49.86 ± 1.52	2.41 ± 0.12
0.08	0.39 ± 0.03	11.33 ± 0.32	50.64 ± 1.61	2.24 ± 0.11
0.10	0.37 ± 0.02	9.48 ± 0.41	50.14 ± 2.06	1.75 ± 0.13

^a^The standard deviation of the properties is based on the statistical data of five cells.

**Table 3:** Photovoltaic properties of QDSCs from J-V curves^a^.

Electrolyte	*V*_oc_(V)	*J*_sc_(mA/cm^2^)	FF(%)	PCE(%)
Without methanol	0.41 ± 0.02	11.68 ± 0.36	49.86 ± 1.52	2.41 ± 0.12
With 30% methanol	0.43 ± 0.02	18.34 ± 0.42	50.86 ± 1.18	4.01 ± 0.16

^a^The standard deviation of the properties is based on the statistical data of five cells.
